# Functional Neurological Disorder and Autism Spectrum Disorder: A Complex and Potentially Significant Relationship

**DOI:** 10.1002/brb3.70168

**Published:** 2024-12-20

**Authors:** Belen Gonzalez‐Herrero, Francesca Happé, Timothy R. Nicholson, Francesca Morgante, Javier Pagonabarraga, Quinton Deeley, Mark J. Edwards

**Affiliations:** ^1^ Departamento de Medicina Universidad Autónoma de Barcelona (UAB) Bellaterra Spain; ^2^ Neurosciences and Cell Biology Institute, Neuromodulation and Motor Control Section St George's University of London London UK; ^3^ Queen's Hospital, Barking Havering and Redbridge University Hospitals Romford UK; ^4^ Social, Genetic and Developmental Psychiatry Centre, Institute of Psychiatry, Psychology and Neuroscience King's College London London UK; ^5^ Neuropsychiatry Research & Education Group, Institute of Psychiatry, Psychology and Neuroscience King's College London London UK; ^6^ Instituto de Investigación Biomédica de Sant Pau Barcelona Spain; ^7^ Centro de Investigación en Red‐Enfermedades Neurodegenerativas (CIBERNED) Madrid Spain; ^8^ National Autism Unit, South London and Maudsley NHS Foundation Trust London UK; ^9^ Department of Clinical and Basic Neuroscience, Institute of Psychiatry, Psychology and Neuroscience King's College London London UK

**Keywords:** autism spectrum disorder, Bayesian brain, clinical overlap, functional neurological disorder, interoception, review

## Abstract

**Introduction:**

Functional neurological disorder (FND) and autism spectrum disorder (ASD) are two complex neuropsychiatric conditions that have been historically classified within psychiatric domains, resulting in a lack of extensive research, insufficient clinical recognition, and persistent societal stigma. In recent years, there has been an increasing recognition among professionals and affected individuals of their possible overlap. This review explores the potential clinical and mechanistic overlap between FND and ASD, with particular attention to shared symptoms across sensory, motor, and psychiatric domains.

**Methods:**

We conducted a narrative analysis utilizing the PubMed, CINAHL, MEDLINE, and ScienceDirect databases from inception to June 2024. The search employed specific MeSH terms related to ASD and FND. Given the limited data availability, we included all relevant articles that explored the potential connections between FND and ASD, focusing on established findings and theoretical hypotheses areas.

**Results:**

Scientific evidence indicates that FND and ASD may co‐occur more frequently than previously acknowledged and with notable overlaps in their clinical presentations and pathophysiology. Theoretical models that have been applied to FND and ASD, such as the Bayesian brain theory and the tripartite model of autism, may provide valuable insights into the intersection of these conditions. Although much of the current evidence remains speculative, it underscores the need for hypothesis‐driven research to investigate these potential connections further.

**Conclusion:**

ASD and FND are heterogeneous conditions that appear to co‐occur in a subset of individuals, with overlapping symptomatology and possibly shared underlying mechanisms. This hypothesis‐generating review emphasizes the need for further research to better understand these links, ultimately aiming to improve clinical recognition and develop targeted interventions that enhance the quality of life for affected individuals.

## Graphical abstract

This review explores the overlap between functional neurological disorder and autism spectrum disorder, highlighting shared issues like chronic pain, sensory sensitivities, and hypermobility. It also examines theoretical models, including the Bayesian brain theory, to offer insights into this intersection. The ultimate aim is to promote knowledge and research into this co‐occurrence to improve the quality of life of affected individuals.

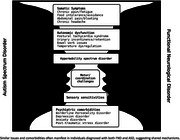

## 1 Introduction

In recent years, data have begun to emerge that link Functional Neurological Disorder (FND) and autism spectrum disorder (ASD), suggesting that these conditions co‐occur more often than expected by chance. However, the opportunity to advance our understanding of FND and ASD is lost because these two spectrum conditions are frequently diagnosed and treated by different medical specialties in adult services, have confusing diagnostic terminology, and are in phases of rapid change in pathophysiological, clinical, and social understanding.

Throughout history, both conditions have endured pervasive stigmatization, been often marginalized within the domains of psychiatric disorders, and have been subject to misconceptions and societal biases. Individuals with FND were often dismissed as feigning symptoms or deemed to have purely psychological ailments, leading to skepticism and invalidation of their experiences. Similarly, autistic individuals faced stigmatization rooted in misconceptions and stereotypes, ranging from being labeled intellectually deficient to being portrayed as lacking emotions. Such stigmatization has comparably led to profound social and emotional challenges for individuals with FND and ASD, hindering their access to appropriate healthcare, educational and work opportunities, and societal inclusion.

FND is defined by neurological symptoms, such as weakness, sensory changes, involuntary movements, gait disturbance, functional seizures (FSs), and speech problems, which have positive clinical features of inconsistency and incongruity with other typical neurological diseases. FND is a common reason for people to seek help from neurological services (Stone et al. 2010). It causes significant disability and poor quality of life (Gendre et al. 2019), and many people remain with symptoms in the long term (Gelauff et al. 2014). Symptom onset is usually in middle adulthood (mean age is 40 years [Perez et al. 2021]), and women are disproportionately more affected than men (2–3:1) at all ages (McLoughlin et al. 2023) (Tinazzi et al. 2020; Baizabal‐Carvallo and Jankovic 2020). Frequently, people with FND also present with functional somatic syndromes, such as irritable bowel syndrome (IBS), fibromyalgia/widespread chronic pain (FM/CWP), or chronic fatigue syndrome (CFS) (Butler et al. 2021). Despite these being listed as separate conditions in DSM‐5, there has been an increasing recognition of overlapping clinical and etiological characteristics between functional somatic symptoms themselves but also with FND (Wessely et al. 1999; Teodoro, Edwards, and Isaacs 2018; Petersen et al. 2020; Yunus 2007).

The etiology of FND is not fully understood. Data suggest various predisposing, precipitating, and perpetuating factors. Previous stressful life events, including childhood or adult‐life abuse, certain personality traits (particularly obsessive–compulsive personality traits), anxiety, and depressive disorders, are significantly more common in patients with FND than healthy and patient controls (Ludwig et al. 2018; Ekanayake et al. 2017). However, such predisposing factors are also absent in many people with FND, leading to unresolved questions about the exact underlying mechanisms causing functional symptoms.

ASD is a phenotypically heterogeneous neurodevelopmental syndrome characterized by persistent difficulties in social communication and interaction alongside restricted and repetitive patterns of behavior, interests, or activities (ICD 11, DSM V). It is believed to have a primarily genetic etiology (Robinson et al. 2011), but environmental and sociocultural factors influence its phenotypic expression (Matson et al. 2017).

ASD, as well as other conditions under the umbrella of neurodiversity, has progressively gained visibility and recognition and is frequently addressed in mainstream media. Parallel to the social interest, there is growing interest among the scientific community, especially around missed or delayed diagnosis and its coexistence with other conditions. ASD is traditionally believed to have a significantly higher prevalence in men than women (4–3:1) (Loomes, Hull, and Mandy 2017). Early identification and support of ASD is crucial for optimizing the outcomes of individuals with the diagnosis. (Rebecca et al. 2014) However, growing research suggests that there may be underdiagnosis or misdiagnosis of autistic females due to various factors, including differences in symptom manifestation, social expectations, masking behaviors, and diagnostic biases based historically on male‐centric presentations of ASD (Bargiela, Steward, and Mandy 2016; Beck et al. 2020). For example, compared to males, autistic females tend to present with more social topics in their special interests and have less pronounced social communication deficits (Wood‐Downie et al. 2021). Despite the obvious advantages of successful camouflaging and social adaptation skills for females, these are likely to come at a cognitive/emotional cost that may contribute to poor mental health. Indeed, autistic females are more likely than autistic males to experience anxiety and depression (Lai et al. 2017; Mandy et al. 2012), and suicide rates are significantly higher (Osman et al. 2001; Oswald et al. 2016).

Autistic individuals often experience challenges related to motor control, sensory sensitivities, and various somatic symptoms, which significantly impact their daily lives. Motor coordination difficulties are common, manifesting as clumsiness, tip‐toe gait, or challenges in fine motor tasks (Fournier et al. 2010). Additionally, sensory sensitivities are prevalent, with individuals experiencing heightened or diminished responses to sensory stimuli, including sound, light, touch, taste, and smell (Robertson and Baron‐Cohen 2017). For instance, they may become overwhelmed by loud noises or find certain textures unbearable. Moreover, many autistic individuals report experiencing somatic symptoms, such as gastrointestinal issues, sleep disturbances, and chronic pain (Hogendoorn et al. 2023).

This review aims to provide a comprehensive understanding of the link between ASD and FND, emphasizing its significance in clinical practice and research. We will begin by exploring the rationale behind studying this association, highlighting the implications for diagnosis, treatment, and theoretical understanding of both conditions. Drawing upon available scientific data, we will examine empirical evidence supporting the co‐occurrence and clinical comorbidities between ASD and FND, shedding light on the nuanced interplay between these disorders. Furthermore, we will delve into theoretical frameworks, including Bayesian brain theories and the tripartite model of autism, to elucidate potential mechanisms underlying the overlap. Through this integrative synthesis, we aim to advance our understanding of the intersecting pathways between ASD and FND, paving the way for more research on the topic and ultimately targeted interventions and improved outcomes for affected individuals.

## 2 Why Understanding the Link Between ASD and FND is Important

The link between ASD and FND has been an increasingly frequent topic for many years in FND patients’ forums, akin to discussions regarding other potentially mechanistically related conditions and symptoms, such as chronic pain. Yet, science has been slow to recognize this potential association, which has only recently generated research interest. This highlights the importance of patient and public involvement when embarking on research.

We acknowledge potential negative consequences of further diagnostic labeling (Coggon, Barker, and Rose 2009; Sims et al. 2021), which may be particularly true in FND as many patients already hold several diagnoses (e.g., FND, FM, fatigue), which arguably may have considerable overlap in pathophysiology. However, if there is an association, identifying it could result in a number of benefits. People with FND often report life‐long challenges in social interaction, struggles with educational attainment, and negative experiences of schooling, which the diagnosis of FND alone cannot easily explain. Similarly, many autistic people have somatic and motor symptoms that are poorly understood within the context of autism itself and, therefore, often overlooked. Recognizing that both conditions may coexist could provide a richer and more complete explanatory framework, opening the door to personalized treatment strategies. At a scientific level, studying the link between ASD and FND could lead to insights into the complex interactions among the brain, body, behavior, psychology, and sociocultural factors. Further research could reveal shared pathways, risk factors, or genetic influences contributing to both conditions that ultimately could lead to therapeutic innovations in both conditions.

Although the current evidence relates mainly to the association between ASD and FND, we recognize that other neurodivergences, such as ADHD, may also overlap with FND and need to be explored.

## 3 Current Estimates of the Prevalence of ASD and FND

The epidemiology of FND presents a complex picture influenced by various factors, such as diagnostic criteria, healthcare settings, and population demographics. Although exact prevalence estimates vary, FND is recognized as relatively common, with some studies suggesting rates ranging from 2 to 30 per 100,000 population (Akagi and House et al. 2001; Selim and Hauser 2000). Comorbidity of FND with other psychiatric and medical conditions is common (Butler et al. 2021; Romero et al. 2016), further complicating the epidemiological landscape.

Regarding ASD, the first studies of the general prevalence conducted in the 1960s and 1970s reported prevalence estimates of 2 to 4 cases per 10,000 children (Lotter 1966; Treffert 1970). This prevalence has steadily risen to current community‐based estimated prevalence rates for ASD in the general population of 0.7%–1.1% (Brugha et al. 2016; Baxter et al. 2015) and even higher within psychiatric inpatient settings ranging from 2.4% to 9.9%, according to available data (Tromans et al. 2018), which is nonetheless scarce and of low quality. The increase in prevalence is mostly due to the expansion of diagnostic criteria and improvements in screening and services for children (Committee to Evaluate the Supplemental Security Income Disability Program for Children with Mental Disorders 2015). Still, a significant number of adults are thought to have undiagnosed ASD (O'nions et al. 2023), especially in more subtle forms, in women (Gesi, Migliarese et al. 2021) and the elderly (O'nions et al. 2023), and a proportion of them have been mistakenly diagnosed with other psychiatric or neuropsychiatric conditions (Bargiela, Steward, and Mandy 2016; Vasiliki, Milou et al. 2021; Wing and Potter 2002; Lai and Baron‐Cohen 2015).

The fact that ASD diagnosis has been more commonly missed in women, whereas FND mostly affects women, may help to explain why it is only in recent years that we increasingly recognize these conditions to coexist in some people. In ASD, delayed diagnosis often occurs due to various factors, including male‐centric diagnostic criteria that may not be representative of ASD in women, limited awareness among healthcare professionals, cultural differences in recognizing developmental milestones, or disparities in access to diagnostic services. Similarly, FND is frequently misdiagnosed or unrecognized, leading to delayed or inappropriate treatment. Moreover, the stigma associated with FND may contribute to reluctance among healthcare providers and patients to consider FND as a potential cause for the symptoms (Edwards, Yogarajah et al. 2023), further prolonging diagnostic processes. Additionally, functional neurological motor issues in a person with ASD might not be diagnosed because they could be seen as behavioral patterns attributed to ASD, missing an opportunity to attempt treatment.

## 4 Evidence on Rates of Comorbidity Between ASD and FND

Evidence on ASD and FND coexistence is emerging but still scarce. Detailed data from available studies, strengths, and limitations are presented in Tables 1 and 2. The research strategy is described in Box 1.

**TABLE 1:** Literature evidence of the link between autism spectrum disorder (ASD) and functional neurological disorder (FND).

Paper (Country)	Population, *N*	Methodology	Findings/Conclusions	Limitations
ASD and Functional seizures (FSs)				
Miyawaki et al. (2016) (Japan, 2016)	Pediatric, 1	Single case report	First report of FS in a child with undiagnosed ASD	Single case report Descriptive design and not generalizable
Katherine A. Jester, Donna L. Londino, Joy Hayman (USA, 2019)	Adolescents and young adults, 1234	Retrospective observational study on the frequency of coexisting codes for ASD and conversion disorder (CD) in the ED department	*N* = 8/1234 (0.6%) cases with ASD and CD coding (all FSs)	Retrospective Descriptive design Lack of control sample Register‐based study; possibility of misclassification Sample possibly non‐representative (single center)
McWilliams A, Reilly C, Gupta J, Hadji‐Michael M, Srinivasan R, Heyman I. (UK, 2019)	Pediatric, 59	Retrospective case series of ASD diagnosis in children referred to a specialist pediatric mental health service	*N* = 10/59 (17%) of the cases with FS also had ASD. *N* = 5/10 (50%) were diagnosed with ASD following the referral	Retrospective Descriptive design Lack of control sample Possibly non‐representative (specialist single center)
Freedman DA, Terry D, Enciso L, Trott K, Burch M, Albert DVF (USA, 2022)	Pediatric ASD, 9 ID, 6	Description of cases with FS and co‐existent ASD or ID	Children with ASD and ID may develop FS and may have good outcomes with early intervention	Retrospective Descriptive design Lack of control sample Small sample, possibly non‐representative (specialist single center)
ASD and FND (type non‐specified)				
Pun P, Frater J, Broughton M, Dob R, Lehn A. (Australia, 2020)	Adolescents and adults with FND, 288	Retrospective observational audit of cases with FND assessed in a specialist clinic	*N* = 28/288 (9%) had a neurodevelopmental condition diagnosed (ADHD, ASD, or intellectual impairment)	Retrospective Descriptive design Lack of control sample Sample possibly non‐representative (specialist single center)
Nisticò V, Goeta D, Iacono A, Tedesco R, Giordano B, Faggioli R, Priori A, Gambini O, Demartini B. (Italy, 2022)	Adults, FND 21, ASD 30, neurotypical 45	Cross‐sectional observational study on the prevalence of self‐reported autistic traits, in people with FND and self‐reported functional symptoms in a cohort of ASD compared to controls Screening tools used: AQ50, RAADS‐R for self‐reported autistic traits and ENQ for self‐reported functional neurological symptoms	The FND group showed no higher AQ or RAADS scores compared to controls The ASD cohort had significantly higher scores in the ENQ compared to controls	Small sample and group numbers are not balanced Sample possibly non‐representative (specialist single center) Findings based on self‐reported screening tools for autistic traits and functional symptoms. No formal clinical assessment was performed
González‐Herrero B, Morgante F, Pagonabarraga J, Stanton B, Edwards MJ (UK, 2022)	Adults with FND, 344	Cross‐sectional observational study (online survey) Prevalence of ASD diagnosis and self‐reported autistic traits and prevalence of ASD diagnosis in their 1st‐degree relative Autistic‐trait screening tool used: AdAS spectrum	*N* = 27/344 (8%) previous diagnosis of ASD *N* = 27/344 (24%) had a 1st‐degree relative with a formal diagnosis of ASD, mostly their children (17%) *N* = 238/344 (69%) of respondents had scores in the AdAS spectrum, indicating a possibly clinically significant ASD *N* = 73/344 (21%) had scores indicating autistic traits	Self‐reported diagnosis of FND Online survey: favors young and computer skills and may fail to reach deprived communities Lack of control sample Charity community (FND Hope), which may not be representative of the broad ASD and FND spectra Screening tools for autistic traits are not substitutes for formal assessments
Cole RH, Elmalem MS, Petrochilos P. (UK, 2023)	Adults with FND,91	Cross‐sectional observational study on the prevalence of autistic traits and alexithymia and its relation with psychiatry comorbidities in a specialized center Autistic‐trait screening tool used: AQ10	*N* = 36/91 (40%) scored positive in the AQ10 and had higher traits for alexithymia, depression, generalized anxiety, social phobia, ADHD, and dyslexia compared to those who scored negative in AQ10	Small sample and possibly non‐representative (specialist single center) Lack of control sample Screening tools for autistic traits are not substitutes for formal assessments

*Note*: *N*, number of people.

Abbreviations: AdAS Spectrum, The Adult Autism Subthreshold Spectrum; AQ10, Autism Spectrum Quotient 10; AQ50, Autism Spectrum Quotient 50; CBT, cognitive behavioral therapy; CD, conversion disorder; ENQ, Edinburgh Neurosymptoms Questionnaire; FND, functional neurological disorder; FS, functional seizure; ID, intellectual disability; RAADS‐R, Ritvo Autism Asperger Diagnostic Scale—Revised; SDQ‐20, Somatoform Dissociation Questionnaire; TAS‐20, Toronto Alexithymia Scale.

**TABLE 2:** Literature evidence of the link between autism spectrum disorder (ASD) and other somatoform disorders.

Paper (Country)	Population, *N*	Methodology	Findings/Conclusions	Limitations
Hatta et al. (Japan, 2019)	Children with SSD, 28 vs. HC, 26	Group comparison analysis of autistic traits in children with SSD vs. HC Autistic‐trait screening tool used: AQ children's version	42.9% of children with SSD had an AQ scored above the average Japanese population, but the between‐group difference was insignificant. For subscale scores, the mean attention switching score of the SSD group was significantly higher in the SSD than in the HC (*p* = 0.005)	Small sample size Findings based on self‐reported measures Screening tools for autistic traits are not substitutes for formal assessments
Asztely et al. (Sweden, 2019)	Autistic females, 77	Prospective longitudinal females diagnosed with ASD and/or ADHD in childhood/adolescence Pain and health‐related measures: SF‐36	76% of the subjects reported chronic pain and a low quality of life	Descriptive design Lack of healthy control group Sample possibly non‐representative
Williams et al. (USA, 2019)	Autistic young adults, 290	A survey in autistic young adults to study somatic symptoms PHQ15; SRS‐II	Higher somatic symptoms compared to the general population previous accounts, mostly sleep issues, menstruation pains, and fatigue	Self‐reported diagnosis of ASD An online survey from an autism charity community may not be representative of the broad ASD spectrum Findings based on self‐reported measures Lack of formal control group
Nimmo‐Smith V, Heuvelman H, Dalman C, Lundberg M, Idring S, Carpenter P, Magnusson C, Rai D (Stockholm, 2020)	Autistic adults, 4049 vs. Non‐autistic adults, 217,645	Retrospective study of all cases registered in the Stockholm Health Cohort and the prevalence of specific anxiety disorders Group comparison analysis of prevalence among autistic adults vs. non‐autistic adults	Relative risk of 3.11 (2.08–4.65) for somatoform disorders in individuals with ASD compared to non‐autistic	Register‐based study; possibility of exposure and outcome misclassification Records from secondary and tertiary care (primary care diagnosis missed)
Zdankiewicz‐Ścigała E, Ścigała D, Sikora J, Kwaterniak W, Longobardi C. (Poland, 2021)	Autistic adults, 79 vs. non‐autistic adults, 126	Group comparison analysis on the prevalence of somatoform disorders Measurement of somatoform disorders using SDQ‐20	Autistic adults presented higher levels of somatoform disorders per SDQ‐20 compared to controls	Findings based on self‐reported measures No access to clinical information Sample possibly non‐representative (charity‐based)
Rødgaard, EM., Jensen, K., Miskowiak, K.W. Mottron, L (Denmark, 2021)	Autistic adults, 16,126 vs. Non‐autistic, 654,977	A retrospective observational study from the Danish National Patient Registry of childhood diagnoses in individuals diagnosed as autistic in adulthood	People diagnosed with ASD in adulthood showed an OR 5.7 (*p* < 0.001) of dissociative/conversion disorders and an OR 2.9 (=*p* < 0.05) of somatoform disorders diagnosis in childhood compared to controls	Register‐based study; possibility of exposure and outcome misclassification Records from secondary and tertiary care. Primary care diagnosis missed Sample possibly non‐representative (single country study)
Larkin et al. (UK and Ireland, 2023)	Autistic adults, suspected autistic adults, 32 Non‐autistic, 119	Online survey, including AQ10, TAS‐20, and PHQ‐15	Known and suspected autistic individuals had higher levels of self‐reported somatic symptoms	Self‐reported diagnosis of ASD Non‐representative sample: mostly white females Online survey: favors young and computer skills and may fail to reach deprived communities Self‐reported tools

*Note*: *N*, number of people.

Abbreviations: AQ10, Autism Spectrum Quotient 10; PHQ‐15, Patient Health Questionnaire‐15; SF‐36, The 36‐Item Short Form Health Survey; SRS‐II, Social Responsiveness Scale II; TAS‐20, Toronto Alexithymia Scale.

Studies have found rates of diagnosed ASD ranging from 8% to 17% (McWilliams, Reilly et al. 2019; Pun, Frater et al. 2020; González‐Herrero, Morgante et al. 2022) and even higher percentages with high autistic traits on self‐report questionnaires (e.g., Autism Spectrum Quotient, The Adult Autism Subthreshold Spectrum) (González‐Herrero, Morgante et al. 2022; Cole, Elmalem et al. 2023). Specifically, our group found in a cohort of 344 people with FND who were self‐selected following an advertisement with an FND Charity, a high prevalence of a previous diagnosis of ASD (8%), and ASD diagnoses in their 1st‐degree relatives (26%), especially their children. Furthermore, 69% of all respondents scored above the suggested threshold for clinically significant ASD on a validated self‐report questionnaire (González‐Herrero, Morgante et al. 2022). Of note, one study showed contrasting data in a cohort of 21 people with FND who did not differ from controls in self‐reported autistic traits (Nisticò, Goeta et al. 2022). All these data have been recently systematically analyzed to conclude that about 10% of children with FSs also have an ASD diagnosis. This same review stated that individuals with ASD are more prone to functional somatic disorders, and ASD rates seem higher in other FNDs like functional motor disorders (Vickers, Menhinnitt et al. 2024).

Some data are also available regarding the presence of functional neurological symptoms in people with a primary diagnosis of ASD. Nisticò et al. (Nisticò, Goeta et al. 2022) found in a cohort of 30 autistic individuals that 87% reported at least 1 functional neurological symptom, a prevalence significantly higher than the neurotypical control group (36%, *p* < 0.001). Another study compared a control sample of 654,977 people with 16,126 individuals identified as autistic in adulthood and found an OR of 5.7 for childhood diagnosis of dissociative/conversion disorders (CD) in the latter (Rødgaard, Jensen et al. 2021). Finally, a retrospective observational study revealed a prevalence rate of concurrent diagnostic codes for ASD and CD within the emergency department setting, amounting to 0.6% (*N* = 8/1234), exclusively characterized by FSs (Jester, Londino et al. 2019). Although this percentage lacks statistical significance and may be attributable to random chance, it represents one of the initial studies acknowledging the potential association between FS and individuals with ASD.

All these data available need to be cautiously interpreted. FND is a neuropsychiatric condition, so as with other neuropsychiatric conditions, higher coexisting ASD prevalence is expected (Tromans et al. 2018). Additionally, many studies described above are mostly retrospective, based on small samples and clinical populations, all subject to important potential bias. Furthermore, most studies based their diagnoses of ASD solely on self‐report scales, which are not a substitute for a formal clinical assessment.

Despite all of the above, rates of ASD are consistently reported higher among males, so the fact that high rates of ASD are being reported in FND, a condition overrepresented in women, makes the likelihood of a true relationship between the two disorders more likely. At the very least, the current data indicate that this area merits further research.

## 5 Symptom and Comorbidity Overlap Between ASD and FND

**FIGURE 1:**
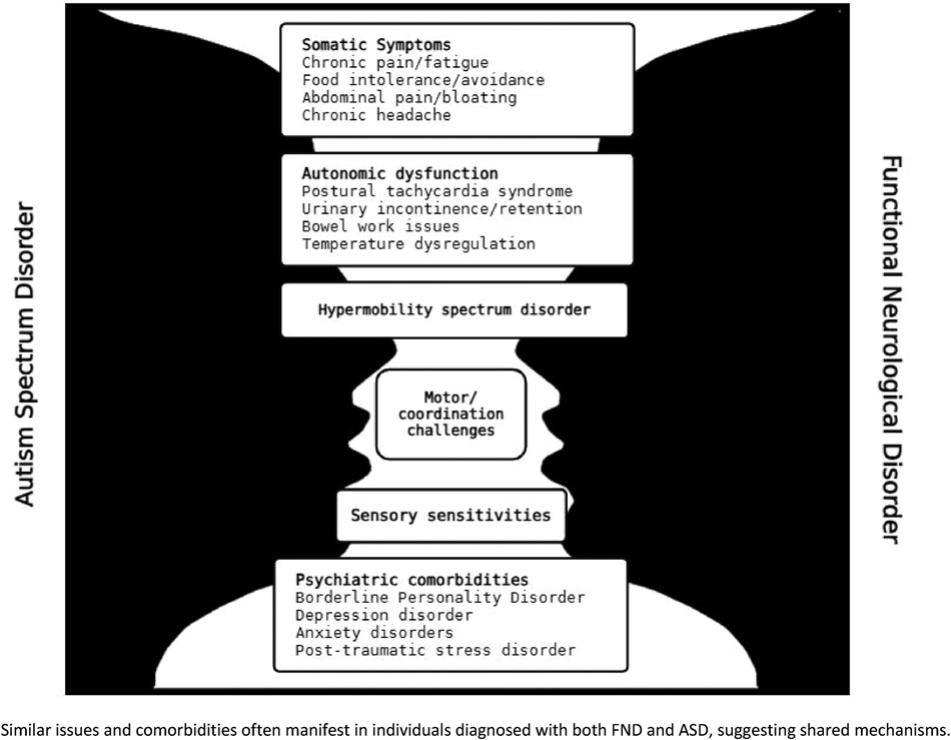
Functional neurological disorder and autism spectrum disorder comorbidity overlap. Similar issues and comorbidities often manifest in individuals diagnosed with both FND and ASD, suggesting shared mechanisms.

Autism is defined by behavioral symptomatology, whereas neurological symptoms and signs with specific positive features (e.g., inconsistency and variability depending on attentional diversion) define FND. Despite their very different diagnostic criteria, they share many symptoms, and comorbidities are worth highlighting (Figure 1); however, we recognize the complexity of the topic and the heterogeneous spectrums along which both conditions present.

### 5.1 Problems With Motor Function

Functional movement disorder (FMD) is a subtype of FND in which the primary manifestations are weakness, tremors, tics, dystonia, gait disorder, or another abnormal movement incongruent with typical neurologic disease but which are nevertheless genuine. The diagnosis of FMD should not be one of exclusion and always rest on clear positive evidence on physical examination, such as Hoover's/abductor's signs used for functional motor weakness, which involves observing contralateral leg movements during hip flexion/abduction against resistance or the “entrainment phenomenon,” where frequency and rhythm of a tremor temporarily synchronize with an external rhythmic stimulus. (Daum, Hubschmid et al. 2014) FMD very often begins abruptly, regardless of phenotype (Perez et al. 2021). Progression to maximum symptom severity and disability is often rapid, which is uncommon in other movement disorders. Illness, surgery, and minor physical injuries are common precipitants.

Impairment in self‐agency, the sense of control of one's actions, is a characteristic feature of FMD (Drane, Fani et al. 2020) believed to be mediated by dysfunction involving a multimodal integration network, including the right temporoparietal junction (TPJ) (Zito, Wiest et al. 2020). Patients with FMD report a lack of voluntary control over their body movements despite physiologic evidence demonstrating that normal movements are possible and often occur when attention is diverted (e.g., cessation of functional tremor with distraction) (Kranick and Hallett 2013; Maurer, LaFaver et al. 2016).

An impaired sense of agency is also present in some autistic people (Zalla and Sperduti 2015), and it is increasingly recognized that some autistic people display pronounced motor impairments in gait and balance, arm function, coordination, and movement planning with frequent freezing (Vilensky, Damasio et al. 1981; Mari, Castiello et al. 2003; Vernazza‐Martin, Martin et al. 2005; Rinehart, Tonge et al. 2006; Esposito and Venuti 2008). However, to date, no evidence exists that these movement abnormalities show function normalization when attention is diverted. Delays in motor milestones have been consistently reported by parents, as well as noted in experimental studies (Harris 2017). More focused studies on the nature of motor impairment in ASD reveal that autistic children may be particularly impaired in tasks that require efficient visual‐motor integration (VMI), such as imitation, which in turn may lead to difficulties in social–communicative skill development (Lidstone and Mostofsky 2021).

Interestingly, as well, some individuals with ASD display catatonic symptoms (Billstedt, Gillberg et al. 2005), a complex syndrome of motor dysregulation (Solmi, Pigato et al. 2018), and cases of “conversion catatonia” are also described in the literature (Jensen 1984; Shah, Meyer et al. 2012; Sallin, Lagercrantz et al. 2016). The DSM‐5‐TR criteria for catatonia include the presence of three clinical features from the following list of 12: stupor; catalepsy; waxy flexibility; mutism; negativism; posturing; mannerisms; stereotypy; agitation; grimacing; echolalia; and echopraxia. Other common signs are motor resistance to simple commands, rigidity, and automatic obedience. However, the definition of catatonia makes its diagnosis challenging in ASD as it captures motor behaviors that can be part of the motor differences in neurodevelopmental disorders. In the seminal paper of Wing et al. (Wing and Shah 2000), catatonia is considered in individuals with autism when they present with (a) increased slowness and parkinsonian features, such as tremors, dystonia, odd stiff posture, and freezing in postures; (b) difficulty in initiating and completing actions; (c) increased reliance on physical or verbal prompting by others; (d) increased passivity and apparent lack of motivation; (e) reversal of day and night; (f) excitement and agitation; or (g) increase in repetitive, ritualistic behavior. Typically, individuals affected are autistic adolescents who find it increasingly difficult to maintain independence due to the onset of a range of chronic motor symptoms. In most affected autistic individuals, catatonic signs start at the ages of 10–19, and slowness, stiff postures, and gait disturbance are the most common presentations (Wing and Shah 2000). There seems to be a gradual presentation of catatonic symptoms in autistic people rather than the full‐blown catatonic stupor state, and there seems to be limited evidence of the effect of benzodiazepines or electroconvulsive therapy therapies (Wing and Shah 2000; Hare, Bunton et al. 2014; Vaquerizo‐Serrano, Salazar De Pablo et al. 2021).

Experts on autistic catatonia consider that undiagnosed ASD should be considered in any teenager presenting with the above‐described symptoms (Shah 2019).

Furthermore, reading the description of catatonia in autism given by Wing et al. (Wing and Shah 2000; Dhossche, Shah et al. 2006), the question arises as to whether what psychiatrists refer to as catatonia‐like deterioration in some autistic people, a neurologist would refer to as FMD. In that case, it would be interesting to test whether attention diversion ameliorates catatonic motor signs in ASD, which, to our knowledge, has not been explored before. This in turn raises the question of whether attention in people with ASD can be as readily diverted as in neurotypical individuals, pointing to a potential role for attentional hyperfocus in people with ASD as a predisposing and maintaining factor for functional symptoms (see below).

### 5.2 Sensory Issues

People with FND frequently report sensory symptoms in all modalities. Sensory processing difficulties in FND include tendencies toward sensory insensitivity in some circumstances (e.g., to pain), sensory sensitivities (e.g., to food textures, lights, and sounds), and sensory avoidance (possibly related to triggering other symptoms) (Ranford, MacLean et al. 2020). Many patients experience combinations of numbness, dysesthesia, and pain. Researchers have explained this by proposing that people with FND have abnormal top‐down predictions about the sensory state of the body that cannot be updated or adjusted by bottom‐up information due to an altered inference system, further perpetuating a vicious circle (Ricciardi, Demartini et al. 2016). These same mechanisms may lie behind placebo and nocebo effects that can be induced transiently in healthy individuals (Fiorio, Braga et al. 2022).

Sensory sensitivities have been a key feature of clinical descriptions of ASD from the original reports by Grunya Sukhareva (Sher and Gibson 2023), Asperger (Asperger 1944), and Kanner (Kanner 1943) to first‐person accounts (Robertson and David 2015). About 90% of ASD individuals self‐report atypical sensory experiences (Pellicano and Burr 2012), encompassing multiple modalities (vision, hearing, touch, olfaction, gustation) (Balasco, Provenzano et al. 2019), to the point that current theories propose that autistic sensory differences may be core phenotypic markers of autism rather than secondary to alterations in neural domains (such as attention) affecting social and sensory processing (Robertson and Baron‐Cohen 2017). In fact, sensory processing differences in autism are visible from early neurodevelopmental stages (Baranek 1999; Rogers, Hepburn et al. 2003; Baranek, Watson et al. 2013) and seem to predict diagnostic status later in childhood (Kaldy, Kraper et al. 2011; Turner‐Brown, Baranek et al. 2013) as well as severity in the social and cognition domains in adulthood (Tavassoli, Hoekstra et al. 2014).

These parallels in sensory issues suggest a potential overlap in the underlying mechanisms of sensory processing abnormalities in ASD and FND that merit further research.

### 5.3 Somatic Symptoms

Beyond the neurological symptoms accounting for their diagnosis, people with FND commonly have high rates of other, often chronic, somatic symptoms, including pain, fatigue, headaches, symptoms in other systems (e.g., cardiac, respiratory, gastrointestinal, and urological), sleep disturbances, or memory difficulties, and these significantly add to lower quality of life and poor treatment outcomes (Bowman and Markand 1996; Dixit, Popescu et al. 2013; Butler, Shipston‐Sharman et al. 2021; Ducroizet, Zimianti et al. 2023).

There is also growing evidence that somatic symptoms (e.g., pain, fatigue, fainting spells, headache, and gastrointestinal complaints) are frequent among autistic individuals (Gurney, McPheeters et al. 2006; Wilson, Manangan et al. 2014; Fulceri, Morelli et al. 2016; Lever and Geurts 2016; Okamoto Y 2017; Asztély, Kopp et al. 2019; Restrepo, Angkustsiri et al. 2020; Mayes, Calhoun et al. 2021; Hogendoorn, Hartman et al. 2023) as well. A survey of 100,000 parents, including the parents of 483 autistic children, reported frequent respiratory issues (24%), food intolerances/avoidance (14%), migraine (12%), and skin allergies (14%), and these rates were significantly higher than for non‐autistic children (Gurney, McPheeters et al. 2006). In 290 autistic young adults, the somatic symptom burden was much higher than previously reported in the general population. The most reported symptoms were fatigue (73%), sleep problems (69%), and menstrual pain (61% of females). Both individual symptoms and total symptom burden were associated with higher levels of depression, anxiety, and autistic traits, along with lower quality of life (Williams and Gotham 2022). In another cohort of 62 autistic teenagers, more than half had had “psychosomatic” symptoms in their childhood; this was significantly more frequent in girls than boys, and those with “psychosomatic” symptoms were more frequently diagnosed in young adulthood with mood disorders, eating disorders, anxiety disorders, or psychotic symptoms (Okamoto Y 2017). In 77 females diagnosed with ASD and/or ADHD in childhood/adolescence, 76.6% reported chronic pain in adulthood and worse quality of life linked to it (Asztély, Kopp et al. 2019). Gastrointestinal symptoms are also frequent; a meta‐analysis found a raised prevalence of these among autistic children (OR, 4.42; 95% CI, 1.90–10.28). Most commonly reported were diarrhea (OR, 3.63; 95% CI, 1.82–7.23), constipation (OR, 3.86; 95% CI, 2.23–6.71), and unexplained abdominal pain (OR, 2.45; 95% CI, 1.19–5.07) (McElhanon, McCracken et al. 2014).

In summary, it seems that in autistic people, somatic symptoms are frequent, arise mostly after puberty, and steadily worsen over time. They are associated with being female and having higher levels of autistic traits, more severe symptoms of anxiety and depression, and a lower overall quality of life (Williams and Gotham 2022). Studies have also found that they correlate with higher interoceptive sensibility (autonomic reactivity) and alexithymia (difficulty identifying own emotions) (Zdankiewicz‐Ścigała, Ścigała et al. 2021).

### 5.4 Autonomic Dysfunction

Autonomic dysfunction refers to a dysregulation of the autonomic nervous system (ANS), which controls various involuntary bodily functions, such as heart rate, digestion, breathing, and temperature regulation. ANS imbalance can result in a wide range of physical health problems, such as breathing difficulties, digestive issues, dizziness, abnormal sweating, or urinary/bowel problems (Cheshire 2012). Several studies have found that autistic individuals and people with FND may experience shared alterations in autonomic function. Postural tachycardia syndrome (rapid increase in heartbeat when changing from sitting or lying down to standing), vasovagal syncope (a drop in heart rate and blood pressure that is triggered by certain environmental or emotional stimuli), dyspnea (difficulties breathing), urinary retention, and constipation are more frequent in autistic people compared to neurotypical individuals (Xue, Peter et al. 2005; William 2012; Gubbiotti, Balboni et al. 2019) and notable in reports of people with FND (Yugué, Shiba et al. 2004; Hill and Haydel 2006; Özsungur, Foto‐Özdemir et al. 2012; Robert Leger 2020; Hoeritzauer, Stanton et al. 2022; Paredes‐Echeverri, Maggio et al. 2022; Sara Paredes‐Echeverri, Julie Maggio et al. 2022). There is an ongoing debate on whether autonomic dysfunction is a feature per se of these conditions or attributable to anxiety, as autonomic symptoms in both conditions show positive correlations with stress, anxiety and panic attacks (Smeekens, Didden et al. 2015; Hoeritzauer, Carson et al. 2021; Taylor, Livingston et al. 2021; Barbier, Chen et al. 2022). Still, some argue that, despite anxiety explaining significant measurable changes in ANS, the autonomic function is still atypical in ASD, presenting sympathetic over‐arousal and parasympathetic under‐arousal compared to otherwise neurotypical individuals with anxiety (Kushki, Drumm et al. 2013) however, this has also been recently challenged (Taylor, Livingston et al. 2021). As far as we know, this has not been specifically studied in FND.

### 5.5 Hypermobility

Hypermobility is the defining characteristic of Ehlers–Danlos syndrome/hypermobile spectrum disorder (EDS/HSD). It refers to increased joint flexibility and range of motion beyond what is considered normal, predisposing to injuries and pain. Although hypermobility is not a defining characteristic of ASD, it has been associated with developmental proprioceptive differences and has been observed to occur more frequently in autistic individuals compared to the general population (Casanova, Sharp et al. 2019; Casanova, Baeza‐Velasco et al. 2020; Glans, Thelin et al. 2021; Eccles, Quadt et al. 2024). EDS/HSD is also significantly more often diagnosed in women versus men (Castori, Camerota et al. 2010); it seems frequent in people with complex chronic conditions such as chronic pain/fatigue (Eccles, Quadt et al. 2024) and is also a frequent diagnosis in a subgroup of FND patients, particularly those presenting with fixed dystonia (Kassavetis, Batla et al. 2012; Rubio‐Agusti, Kojovic et al. 2012; Delgado, Kurtis et al. 2022). Nisticò et al. (Nisticò, Iacono et al. 2022) also found that a small cohort of autistic people and people with FND both had more EDS/HSD‐related symptoms than the general population (see Table 1 for details).

### 5.6 Psychiatric Comorbidity

Studies of adults with FND have reported a prevalence of psychiatric disorders in 51% (Thomas, Vuong et al. 2006) to 95% (Feinstein, Stergiopoulos et al. 2001), and this seems to be higher in people presenting with FSs than FMD (Patron, Rustomji et al. 2022). The most common psychiatric comorbidities include depression, generalized anxiety disorder, and post‐traumatic stress disorder (PTSD), with prevalence rates ranging from 21% to 48% (Paredes‐Echeverri, Guthrie et al. 2022; Patron, Rustomji et al. 2022). Panic disorder and obsessive‐compulsive disorder (OCD) are also observed, albeit less frequently (Goldstein, Robinson et al. 2020). Personality disorders are prevalent in over half of adults with FND, including emotionally unstable personality disorder (EUPD) (Feinstein, Stergiopoulos et al. 2001). Conditions like eating disorders, psychotic illnesses, and substance use disorders seem rare (Patron, Rustomji et al. 2022).

In regards to ASD, research has yielded a broad spectrum of prevalence rates for psychiatric comorbidities. This may partly reflect problems with symptom differentiation and overshadowing (e.g., obsession and compulsions vs. restricted and repetitive interests), combined with the importance of self‐report in psychiatric diagnosis in a population where alexithymia, literal interpretation of language, and difficulties discerning communicative intention are common (RCP 2020). Anxiety and depression disorders have been reported as ranging between 2% and 50%; OCD and bipolar disorder have been found in 6%–22% of cases, and schizophrenia spectrum and other psychotic disorders have exhibited rates widely ranging from 4% to 67% (Hossain, Khan et al. 2020). There is also some preliminary evidence of an increased prevalence of ASD diagnosis and traits among individuals diagnosed with eating disorders (Huke et al. 2013) and personality disorders, including EUPD (Rydén, Rydén et al. 2008; Dell'Osso, Cremone et al. 2018) and OCPD (Gillett, Leeves et al. 2023). In particular, the relationship between ASD and EUPD is an ongoing area of research and debate (May, Pilkington et al. 2021). Genetic, environmental, and neurobiological factors influence both conditions (Rigles 2017; Witt, Streit et al. 2017; Kulacaoglu and Kose 2018), and they share some features, such as difficulties with emotion regulation, identity and self‐image disturbances, interpersonal challenges, impulsivity and early‐life adversity, and traumatic experiences. The symptomatic overlap of ASD and personality disorders can lead to differential diagnostic uncertainty, particularly in women (Hofvander, Delorme et al. 2009; Tove, Maria Unenge et al. 2012; Lai and Baron‐Cohen 2015), and it may be that in some females, the diagnosis of EUPD overshadows ASD (Dudas, Lovejoy et al. 2017).

Traumatic experiences in childhood and PTSD are frequent among autistic people (Kerns, Newschaffer et al. 2015; Rumball, Happé et al. 2020), and there is likely to be a complex interplay between early trauma and the development of certain personality traits and behaviors, including in those who may be diagnosed with ASD later in life. On the other hand, being autistic can itself sometimes mean being more vulnerable to having adverse experiences (e.g., bullying at school). These experiences may further disrupt an individual's ability to regulate emotions effectively and may add to severe and persistent PTSD or complex PTSD (a broader range of symptoms compared to PTSD, including disturbances in self‐organization, such as negative self‐concept, interpersonal disturbances, and affect dysregulation).

### 5.7 Neurological Comorbidity

Neurological comorbidities are frequently observed in both FND and ASD. Individuals with functional symptoms can present with coexisting neurological conditions such as migraine, multiple sclerosis, epilepsy, stroke, or Parkinson's disease (Stone, Carson et al. 2012; Tinazzi, Geroin et al. 2021; Carle‐Toulemonde, Goutte et al. 2023). In ASD, epilepsy is notably prevalent, especially in those with more severe presentations, with rates ranging from 20% to 30% (Bolton, Carcani‐Rathwell et al. 2011). The co‐occurrence of epilepsy and FSs is well documented (Asadi‐Pooya and Farazdaghi 2021), and this overlap has also been reported in ASD (Miyawaki, Iwakura et al. 2016; McWilliams, Reilly et al. 2019).

## 6 Neuroimaging Findings in ASD and FND

The broad spectrum of ASD and FND and, in general, small patient cohorts in previous studies complicates the interpretation of research into structural and functional brain abnormalities in these conditions. Systematic reviews and meta‐analyses of neuroimaging in these conditions have yielded conflicting results (Müller, Shih et al. 2011; Nair, Keown et al. 2014; Bègue, Adams et al. 2019; Schielen, Pilmeyer et al. 2024), which may be attributed to several factors. A significant complication is the substantial overlap in brain regions implicated in ASD, FND, and other often co‐morbid neuropsychiatric disorders, such as depression, anxiety, and PTSD (Lai, Kassee et al. 2019; Patron, Rustomji et al. 2022). This overlap makes it difficult to discern whether the observed abnormalities are related specifically to ASD/FND or comorbid conditions. The challenge is exacerbated by the fact that control groups in these studies often consist of healthy/neurotypical individuals rather than clinical populations (King, Prigge et al. 2019). Furthermore, much of our understanding of the neuroanatomical basis of ASD is derived from neuroimaging studies that predominantly examine male participants (Lai et al. 2019), whereas FND occurs more frequently in females (McLoughlin et al. 2023; Tinazzi et al. 2020; Baizabal‐Carvallo and Jankovic 2020). Additionally, previous meta‐analyses of ASD have included participants across a wide age range, which may be a limiting factor. Neuroanatomical and connectivity abnormalities in ASD vary with age (Donovan and Basson 2017), and although FND can occur in children, it is primarily an adult disorder, with most neuroimaging studies focusing on adult cohorts.

Despite these challenges, certain brain regions consistently show abnormalities in both conditions, warranting further investigation. Emerging evidence suggests that the structure, functional activation, and connectivity of brain areas involved in social cognition in ASD may be altered (Pelphrey, Shultz et al. 2011; Deshpande, Libero et al. 2013; Patriquin, DeRamus et al. 2016). These regions, which mediate functions such as attention, agency detection, and emotional perception, include the orbitofrontal cortex, amygdala, TPJ, and superior temporal sulcus (Adolphs 2009). There also seems to be increased connectivity in sensory networks in those individuals with ASD with heightened sensory sensitivity (Marco, Hinkley et al. 2011).

In FND, gray matter alterations in similar areas, including the sensorimotor and cingular–insular regions, as well as increased volume in regions like the amygdala, thalamus, and cerebellum, alongside cortical thinning and white matter disruptions in motor pathways, have been identified (Labate, Cerasa et al. 2012; Aybek, Nicholson et al. 2014; Nicholson, Aybek et al. 2014; Perez, Williams et al. 2017; Maurer, LaFaver et al. 2018). Resting‐state fMRI studies in FND demonstrate aberrant connectivity between emotional processing areas and motor control networks and reduced connectivity between the right TPJ and sensorimotor regions. Task‐based fMRI reveals heightened limbic and paralimbic activity during emotional tasks, with inconsistent patterns of amygdala activation (Voon, Brezing et al. 2010; Aybek, Nicholson et al. 2015; Balachandran, Goodman et al. 2021).

In conclusion, both ASD and FND seem to have notable structural and functional abnormalities, particularly in brain regions linked to social cognition and sensory‐motor integration. The inconsistencies in findings likely result from methodological, analytical, and clinical heterogeneity across studies and affected individuals. Addressing these variations is essential for advancing our understanding of the neurobiological underpinnings of these conditions.

## 7 The Bayesian Brain: A Common Theoretical Framework for Understanding ASD and FND

The Bayesian brain theory is a model that proposes how the brain may process and represent information based on probabilistic inference and Bayesian principles. It suggests that the brain combines prior knowledge or expectations with incoming sensory information to make inferences and guide behavior in a given context (Knill and Pouget 2004).

Although the Bayesian brain theory has been applied to various domains, including perception (Hohwy 2017), decision‐making (Dayan and Daw 2008), and learning (Jacobs and Kruschke 2011), its relevance to psychiatric conditions, neurodiversity, and FND is a topic of ongoing research and debate. Researchers have proposed that atypicality in Bayesian processing could contribute to the cognitive and perceptual characteristics observed in autistic individuals (Pellicano and Burr 2012; Noel, Shivkumar et al. 2022) and symptom generation in people with FND (Edwards, Adams et al. 2012).

We acknowledge that attempts to comprehensively explain the entire spectrum of characteristics observed in ASD and FND with a single unifying theory risk diminishing their diversity and complexity. However, some common characteristics found in ASD and FND are worth mentioning.

### 7.1 Concepts on Predictive Coding, Interoception, and Emotions

Predictive coding is a computational framework rooted in Bayesian principles. It suggests that the brain uses internal generative models acquired through experience or mental simulation to continuously generate descending (top‐down) predictions of expected sensory data, which, to a variable extent, can modulate or gate incoming sensory information (Jacobs and Kruschke 2011). These top‐down predictive signals from higher order brain structures continually update (learning) to minimize discrepancy with incoming ascending (bottom‐up) sensory signals to build a representation of the “world” (Friston, Daunizeau et al. 2010). Sometimes, even without direct access to what is causing the registered sensory information, meaning is obtained from past events that seem similar to the current state of the body and environment (Radulescu, Shin et al. 2021), a process called causal inference. This ability to infer which hidden environmental structure may have generated observed sensory signals is essential to behaving adaptively (Shams and Beierholm 2022). These sensory signals are not solely exteroceptive sensations from the external environment but also registrations of the body's internal state (Seth, Suzuki et al. 2012), a sense called interoception.

Interoception is the process through which internal bodily sensations are processed and through which they may direct behavior and sometimes result in a consciously perceived state, for example, hunger or thirst (Craig 2002). It contributes to maintaining allostasis (Quigley, Kanoski et al. 2021) and provides a sense of one's physical condition (Craig 2002). Interoceptive regulation refers to an individuals’ capacity to notice, categorize, interpret, and respond to interoceptive signals (Joshi, Graziani et al. 2021). It is a multidimensional construct, including interoceptive accuracy, insight, and sensitivity (Millman, Eleanor et al. 2023). Interoception is impacted by attention toward or away from stimuli in a given moment (Petzschner, Weber et al. 2019), which itself depends on the psychological and cultural context in which the individual perceives and processes stimuli and personal confidence in these bodily signals. An example of its function would be disattending to unexpected physical sensations rather than them being appraised as threatening (Farb, Daubenmier et al. 2015).

A growing body of literature exists on how interoception is central to individual differences in emotional awareness and regulation (James 2015; Craig 2002; Barrett 2017). Indeed, interoception training is used to help regulate negative emotions (Füstös, Gramann et al. 2012; Pollatos, Matthias et al. 2015). Traditionally, affective interoception impairment (difficulties identifying, understanding, and expressing one's emotions) has been psychologically described with the term alexithymia (López‐Muñoz and Pérez‐Fernández 2019). However, interoception acts as a bridge between physiological sensations and emotional awareness (Jungilligens, Paredes‐Echeverri et al. 2022). Suppose one has difficulties perceiving and understanding the bodily changes associated with emotions. In that case, one might also struggle to identify and label one's feelings, leading to higher scores on scales designed to assess alexithymia. One might confuse emotional arousal with non‐affective interoceptive states, such as tiredness or a general sense of being unwell. This is further supported by research showing that some people with alexithymia are less accurate than those without this trait in heartbeat perception tasks (Herbert, Herbert et al. 2011; Shah, Hall et al. 2016) or are late to seek medical assessment in response to acute myocardial infarction (Carta, Sancassiani et al. 2013). In line with this, instead of being considered a deficit in affective interoception alone, alexithymia may be one manifestation of a global failure of interoception (Brewer, Cook et al. 2016).

### 7.2 Evidence of the Above Mechanism Alterations in ASD and FND

One can understand how dysfunction at any level, whether it is in exteroceptive sensory registration, interoception, emotions, cognitive priors, learning abilities, or attention, may greatly affect the active inference feedback loop of the Bayesian brain and jeopardize an individual's relation with and representation of the world and their own body.

In FND, (Edwards et al. 2012) first suggested that functional symptoms may arise from internal representations of illness that align with the individual's clinical symptoms, often influenced by past adversities. These representations (cognitive priors) play a significant role in directing attention and consciousness. Therefore, prior beliefs seem to be altered in some people with FND and may be difficult to update by bottom‐up sensory information. Indeed, in FND, contextual factors (the person's sociocultural and psychological states, surrounding available information, and prior knowledge/experiences, which impact how we perceive and make inferences about the world) can influence symptom expression and variability. For example, parental reinforcement of children's illness behavior influences those children's concept of their illness, often resulting in beliefs and symptoms incongruent with their actual state of health, which may persist into adulthood (Benjamin and Eminson 1992). News media, television, and movies are other common sources of exposure to concepts of diseases. Media coverage of a disorder has been associated with an increased presentation to physicians with concerns about that disorder (Stewart 1990) (Frey, Black et al. 2022). These personal and peripheral experiences of illness help shape beliefs about physical symptoms and health and may lead to symptom generation and monitoring.

Additionally, it seems that in some people with FND, the registration of external and internal sensory stimuli is altered (top‐up regulation), which further compromises the system. Knowledge of this topic is evolving and is sometimes contradictory, with discrepancies between studies likely due to individual differences, highly heterogeneous clinical populations, small samples, comparisons only made with healthy control groups, and difficulties with measurement tasks and their interpretation. Cardiac interoceptive accuracy (objective performance monitoring changes in internal body states [Khalsa, Adolphs et al. 2018]) has been found to be reduced in FND compared to healthy controls in some studies (Ricciardi, Demartini et al. 2016; Demartini, Volpe et al. 2019; Koreki, Garfkinel et al. 2020; Williams, Reuber et al. 2021) but not in others (Jungilligens, Wellmer et al. 2020; Pick, Rojas‐Aguiluz et al. 2020). Interoceptive sensibility (the person's perception of their tendency to register internal bodily states in daily life) (Khalsa, Adolphs et al. 2018) has also been found to be heightened (Koreki, Garfkinel et al. 2020) or reduced (Pick, Rojas‐Aguiluz et al. 2020; Ricciardi, Nisticò et al. 2021) in an FND sample compared to healthy controls. There are also conflicting findings on interoceptive awareness (Garfinkel, Seth et al. 2015) (confidence‐accuracy correspondence), with FND samples showing enhanced (Ricciardi, Demartini et al. 2016) or reduced awareness (Millman, Short et al. 2023).

With regards to ASD, research in the sensory domain is also somewhat inconsistent. Some studies have suggested that individuals with ASD have a greater a priori probability of combining audio and visual cues, regardless of whether these signals ought or ought not to be integrated (e.g., they are more likely to perceive asynchronous events as synchronous) (Stevenson, Siemann et al. 2014; Noel, De Niear et al. 2017). This appears to be due to impairment in the causal inference process and, subsequently, difficulties in establishing and updating an internal model that links sensory observations to hidden causes (Noel, Shivkumar et al. 2022; Noel and Angelaki 2023). People with ASD have many sites of differences in processing across the sensorimotor‐association cortical hierarchy, which may contribute to abnormally precise priors which are inadequately updated. This can explain the inflexible updating of expectations and prior beliefs despite new evidence reported in autism (Uddin 2021; Vishne, Jacoby et al. 2021; Noel, Shivkumar et al. 2022; Schneebeli, Haker et al. 2022). Again, although altered audiovisual synchrony perception can happen in some autistic people, a recent study has challenged the fact that there is a difference compared to non‐autistic adults (Weiland, Polderman et al. 2023).

In regards to interoception in ASD, a recent meta‐analysis on interoception abilities (Williams, Suzman et al. 2023) concluded that autistic people perform worse than neurotypical control participants in an objective interoceptive accuracy task (significantly reduced heartbeat counting performance); however, they are significantly more confident in their good performance despite this being worse on average (higher confidence in their heartbeat counting abilities) compared to controls.

Lastly, individuals with ASD and FND often experience heightened anxiety levels, and this can be associated with difficulties tolerating uncertainty (Sandhu, Xiao et al. 2023). Anxiety may lead to overestimating the likelihood of negative outcomes, impacting the accuracy of predictions and influencing decision‐making (Soshi, Nagamine et al. 2019) and symptom maintenance. Additionally, atypical interoception seems to be ubiquitous across neuropsychiatric conditions (Brewer, Murphy et al. 2021). Therefore, it would be key in further understanding the role of altered interoception in ASD and FND for future studies to increase the sample size and to include clinical comparison groups with relevant mental health disorders, such as anxiety, PTSD, depression, or other chronic physical health disorders.

## 8 The Tripartite Framework for Autism and Its Potential Application to People With FND

A novel view on autism proposes a tripartite dimensional framework to explain the broad autism spectrum (Sarovic 2021). This hypothesis suggests that to develop a behavioral phenotype that may be identified as impairing and therefore warranting a diagnosis of ASD, an interaction among three factors is needed: (a) an endophenotypic autistic personality, (b) difficulties with cognitive compensation, and (c) neuropathological burden.

The first dimension, autistic personality, describes certain personality traits such as a tendency toward following an established routine, difficulties with social interactions and non‐verbal communication, or an intense connection with specific ideas or objects. The second proposed dimension is cognitive compensation, which protects against phenotypic maladaptation if adequate but may be taxing. The concept of compensation refers to how an intact neurocognitive process/system might take over, or “compensate” for, the impaired functioning of a faulty process/system in order to maintain typical behavior and/or cognitive task performance (Ullman and Pullman 2015). Compensating, although it helps a person with autistic traits to navigate the world, can also imply that a secondary, perhaps less‐suited cognitive resource is being used, given the absence/malfunctioning of the system that would typically serve the specific purpose (Livingston and Happé 2017). In line with this, compensatory attempts take a toll and contribute to poor mental health in autism (Hull, Petrides et al. 2017; Livingston, Shah et al. 2019). The third dimension, neuropathological burden, refers to the total effect of different insults and their effect on brain development and the ability to maintain allostasis. These insults extend from genetic alterations to infections, toxin exposures, stress, injuries, and traumatic experiences, among others.

It is common that in people with FND, the onset of symptoms happens in adulthood following exposure to a significant emotional or physical stressor (e.g., combat and noncombat‐related accidents, medical illness, surgeries, and work‐related stress) (Linden 2020; Phillips 2021), and in many, there is a significant history of previous adverse life experiences depression, and anxiety (Patron, Rustomji et al. 2022). It may be true for some people with FND that they have an endophenotypic autistic personality but have cognitively compensated for their intrinsic difficulties for years at the expense of a significant psychological burden. Hence, once they develop functional symptoms in a particular context, they may be more likely to get stuck due to the previously described shared mechanism of altered sensory processes, difficulties with interoception, cognitive priors, or attention atypicality.

The population variation in ASD occurrence is strongly related to genetic influences, with an estimated heritability of 80% (Bai, Yip et al. 2019), and studies have found compensated autistic traits in parents of autistic children (Lyall, Constantino et al. 2014). The idea of a compensated autistic personality could partly explain why our group found in a cohort of people with FND (90% women) high rates of diagnosed autistic children (17%) and siblings (6%) but not an ASD diagnosis on themselves despite high self‐reported autistic traits (González‐Herrero, Morgante et al. 2022). The absence of a formal ASD diagnosis may also reflect missed diagnosis, as well as the high sensitivity but poorer specificity of self‐reported screening tools.

Some may question this idea of an intrinsic autistic personality in people with FND but propose a potentially acquired autistic phenotype secondary to early life traumatic experiences, which are frequent in this population. However, trauma, especially in early life, is a known risk factor for developing behaviors that could appear autistic, such as social isolation or difficulties with emotional regulation and communication (Stavropoulos, Bolourian et al. 2018; Saqui, Garvin et al. 2022); people with an autistic personality also seem at greater risk of suffering trauma (Berg, Shiu et al. 2016; Maïano, Normand et al. 2016; Kerns, Newschaffer et al. 2017). Disentangling correlation from causation is particularly difficult in this topic. As ASD tends to run in families, further studies that include data on premorbid and parental autistic personality are needed, as those considered to have “acquired autism” secondary to trauma may have already had an inherited increased liability for an autistic diagnosis or a compensated autistic personality.

An alternative possibility is that people with FND may share some characteristics with people with ASD that predispose to functional and somatic symptom formation while not displaying enough features of ASD, such as problems with reciprocal social interaction and social communication, restricted interests, and sensory processing differences to satisfy diagnostic criteria or be considered to display an autistic personality or endophenotype. Relevant characteristics that may be variously present in people with ASD and FND may include cognitive rigidity and attentional focus (contributing to strongly weighted priors), enhanced affective and autonomic arousal (contributing to salience of cognitive and sensory representations and learning consolidation), alexithymia, altered interception, and sensory processing differences (conducing to misappraisal of either unusually salient or muted sensory information). All of these processes can potentially alter causal inference (perception) of bodily sensations across the sensorimotor‐association cortical hierarchy (Penny, Stephan et al. 2004; Edwards, Fotopoulou et al. 2013).

## 9 Recommendations for Future Research

This review aimed to create a comprehensive understanding of the co‐occurrence of FND and ASD. Upon analysis, it became evident that the research in this area is still in its nascent stages. Still, evidence suggests heightened rates of ASD among those with FND and the presence of “unexplained” somatic symptoms in ASD populations. Large‐scale epidemiological studies are needed for accurate prevalence estimates of both disorders among each other. This will help establish the true extent of the overlap between the two disorders and identify demographic and clinical factors associated with their coexistence. Methodological improvements are essential, such as standardized diagnostic criteria, larger samples, and longitudinal studies to track the development and progression of ASD and FND symptoms over time and identify potential causal relationships to the co‐occurrence of these conditions. Transdiagnostic approaches should explore common underlying mechanisms and vulnerabilities shared by ASD and FND. By examining overlapping neurobiological, psychological, and environmental factors, researchers can gain insights into potential targets for intervention and treatment. Additionally, mechanistic studies using advanced techniques can elucidate neural circuits, genetic factors, and physiological processes underlying their co‐occurrence. Lastly, more intervention research is needed to develop and evaluate tailored interventions addressing the unique needs of individuals with comorbid ASD and FND, including emotional regulation, sensory processing, and functional impairment strategies.

## 10 Conclusions

ASD and FND seem to coexist in a proportion of people. Although there is considerable heterogeneity in people with FND in terms of symptoms, comorbidities, and pre‐illness life events, we believe there are sufficient data to acknowledge that a population with autistic‐like behaviors/characteristics exists among people with FND and merits further research.

We neither present the Bayesian brain framework as the dogma in understanding these conditions nor believe that only one theory can model complex and multifactorial disorders such as FND and ASD. Yet, we think it is a valid and useful model to understand mind–body interactions and how life experiences can affect the way our brain senses our internal and external world.

Understanding the role of neurodiversity in FND and the possibility of FND developing in someone with an established diagnosis of autism could foster a more inclusive and holistic understanding of the range of difficulties some patients face and help develop more individualized, patient‐centered approaches to diagnosis and treatment, recognizing each person's unique experiences, strengths, and challenges. It also highlights the importance of multidisciplinary care that integrates neurological, psychiatric, and rehabilitative perspectives to address the complex interactions between neurological functioning, psychological factors, and social contexts.

## Author Contributions

**Belen Gonzalez‐Herrero**: Conceptualization, investigation, methodology, writing–review and editing, writing–original draft, Data curation. **Francesca Happé**: Writing–review and editing, supervision. **Timothy R Nicholson**: Writing–review and editing, supervision. **Francesca Morgante**: Writing–review and editing, supervision. **Javier Pagonabarraga**: Writing–review and editing, supervision. **Quinton Deeley**: Writing–review and editing, supervision. **Mark J. Edwards**: Conceptualization, investigation, methodology, writing–review and editing, supervision.

## Ethics Statement

The authors have nothing to report.

## Conflicts of Interest

M.E. and T.R.N provide expert medicolegal testimony for people with neurological and psychiatric conditions, including FND. The rest of the authors declare no conflicts of interest in the current study.

### Peer Review

The peer review history for this article is available at https://publons.com/publon/10.1002/brb3.70168.

## Data Availability

Data sharing is not applicable to this article as no new data were created or analyzed in this study.
